# Supplementary material for: Extracellular traps from activated vascular smooth muscle cells drive the progression of atherosclerosis
Source: Nat Commun. 2022 Dec 6;13:7500. doi: 10.1038/s41467-022-35330-1 (PMC9723654; doi:10.1038/s41467-022-35330-1)
Supplement: Supplementary file 1 — Supplementary Information [file 41467_2022_35330_MOESM1_ESM.pdf]

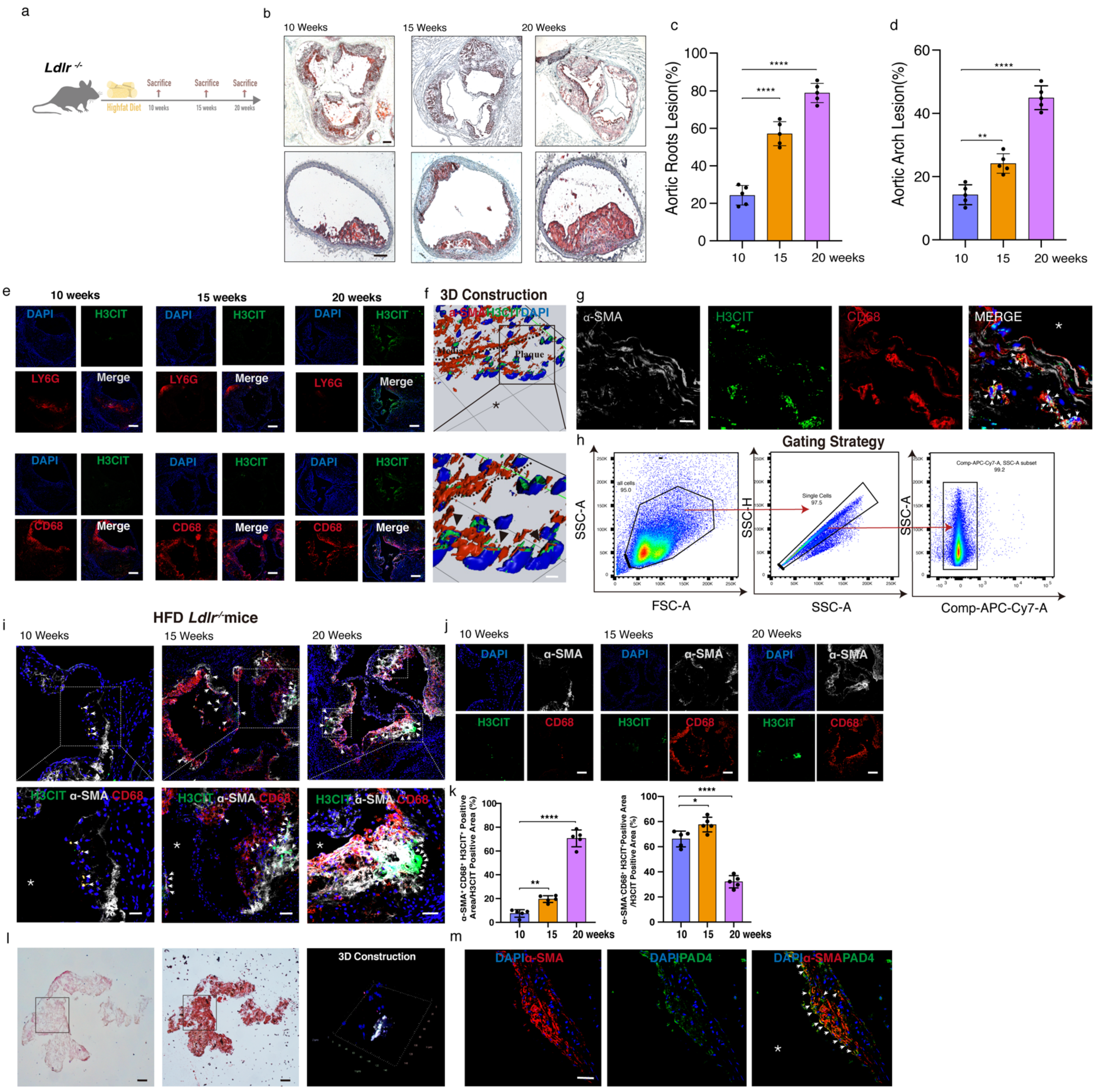

a
Ldlr -/-
Sacrifice
Highfat Diet
10 weeks
15 weeks
20 weeks
b
10 Weeks
15 Weeks
20 Weeks
c
Aortic Roots Lesion(%)
10
15
20 weeks
d
Aortic Arch Lesion(%)
e
10 weeks
15 weeks
20 weeks
DAPI
H3CIT
LY6G
Merge
CD68
f
3D Construction
g
α-SMA
H3CIT
CD68
MERGE
h
Gating Strategy
SSC-A
FSC-A
SSC-H
Comp-APC-Cy7-A
i
HFD Ldlr-/- mice
10 Weeks
15 Weeks
20 Weeks
H3CIT α-SMA CD68
j
k
α-SMA+ CD68+ H3CIT+ Positive Area/H3CIT Positive Area (%)
α-SMA-CD68+ H3CIT+Positive Area /H3CIT Positive Area (%)
l
3D Construction
m
DAPIα-SMA
DAPIPAD4
DAPIα-SMAPAD4

Supplementary Figure 1. Representative Images of Histological Experiments, IF Staining, Gating Strategy of Atherosclerosis Plaque from *Ldlr*$^{-/-}$ mice fed HFD and Patients, Related to Figure 1.

a, Schematic diagram of *Ldlr*$^{-/-}$ mouse fed different timepoint of HFD; **b,** Oil red O staining of the aortic root or aortic arch, scale bar = 100 μm. **c&d** The ratio of plaque lesion area in the total roots area in b, Values were from 5 animals in each group. c (****p < 0.0001) d (**p = 0.0011, ****p < 0.0001). **e,** The single fluorescence and merge image of Figure 1A, scale bar = 100 μm. **f,** 3-dimensional construction IF staining of α-SMA and H3CIT in aortic root plaque. Scale bar = 10 μm. Black triangle symbols show α-SMA$^+$ H3CIT$^+$ cells. **g,** IF staining of the α-SMA, H3CIT, and CD68 in the aortic root plaque. Scale bar = 25 μm; **h,** Supplementary gating strategy for the flow cytometry in Figure 1. **i,** IF staining of the α-SMA, H3CIT, and CD68 in the aortic root plaque sections of different weeks of HFD-fed *Ldlr*$^{-/-}$ mice, scale bar = 25 μm. **j,** The single fluorescence and merge image of Supplementary Figure 1I. scale bar = 100 μm. **k,** The ratio of H3CIT$^+$ CD68$^+$ α-SMA$^+$ area (**p = 0.0034, ****p < 0.0001) or the H3CIT$^+$ CD68$^+$ area (*p = 0.0148, ****p < 0.0001) in the total H3CIT$^+$ area within aortic root plaque(each time point n=5 mice). **l,** The HE-staining or oil red O staining of the human aspiration plaque and 3D construction of IF staining results in H3CIT and DAPI. Scale bar = 100 μm. **m,** IF staining of the PAD4, α-SMA in the sections of aortic arch plaque, scale bar = 25 μm. The side of the white star represents the lumen side. White arrows point at the positive cells within the plaque. For all panels, error bars represent SD. p value was determined by one-way ANOVA with Bonferroni post-test (c,d,k). Each experiment was repeated independently 3 times for (e,g,i,m).

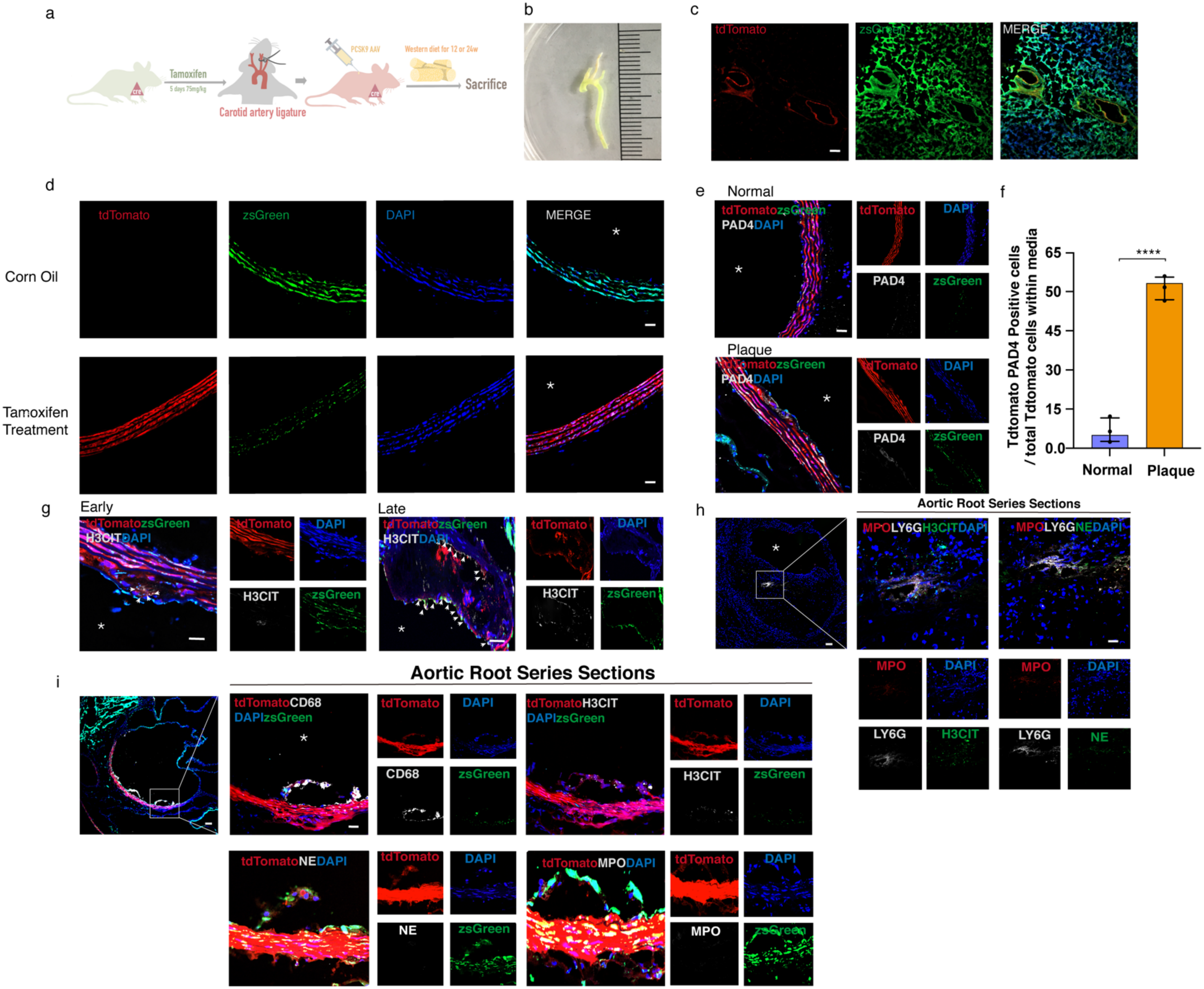

a
Tamoxifen
5 days 75mg/kg
cre
Carotid artery ligature
PCSK9 AAV
Western diet for 12 or 24w
Sacrifice
b
c
tdTomato
zsGreen
MERGE
d
tdTomato
zsGreen
DAPI
MERGE
Corn Oil
Tamoxifen Treatment
e
Normal
Plaque
tdTomatozsGreen PAD4DAPI
tdTomato
DAPI
PAD4
zsGreen
f
Tdtomato PAD4 Positive cells / total Tdtomato cells within media
65
50
45
30
15
0.0
****
Normal
Plaque
g
Early
Late
tdTomatozsGreen H3CITDAPI
tdTomato
DAPI
H3CIT
zsGreen
h
Aortic Root Series Sections
MPOLY6GH3CITDAPI
MPOLY6GNEDAPI
MPO
DAPI
LY6G
H3CIT
NE
i
Aortic Root Series Sections
tdTomatoCD68 DAPIzsGreen
tdTomato
DAPI
CD68
zsGreen
tdTomatoH3CIT DAPIzsGreen
H3CIT
tdTomatoNEDAPI
NE
tdTomatoMPODAPI
MPO

Supplementary Figure 2. Representative Images of IF Staining of Atherosclerosis Plaque Sections in VSMCs-Lineage Tracing Mice, Related to Figure 1.

a, Schematic diagram of VSMCs lineage-tracing mice atherosclerotic model. To track ETs^+ $CD68^+$ VSMCs, we generated a VSMCs-lineage tracing murine model *B6-G/R* $Myh11^{Cre}$. In this model, VSMCs and their progenies permanently expressed Tdtomato after being induced by tamoxifen. To induce plaque formation, we ligated the carotid artery, injected PSCK9-AAV, and fed mice with HFD for 12 or 24 weeks. **b,** The picture of the aorta gross specimen separated from atherosclerotic models. **c,** Showed $Tdtomato^+$ labeled VSMCs of the aorta within the spleen, Scale of bar = 100 µm. **d,** Showed $Myh11^{Cre}$ VSMCs cells will switch from $ZsGreen^+$ to $Tdtomato^+$ labeling after tamoxifen-induced, Scale bar = 20 µm. **e,** Showed PAD4 IF staining on ascending aorta with or without plaque, harvested from HFD-fed mice or control mice, respectively. Scale bar = 50 µm. **f,** The Quantification of the percentage of the number of $PAD4^+$ $Tdtomato^+$ cells in the number of total $Tdtomato^+$ cells between 2 groups (each group n=3 mice). ****$p < 0.0001$. **g,** Location of $Tdtomato^+$ ETs^+ area by staining H3CIT on sections of plaque in ascending aorta of both time points, respectively. Scale bar = 20 µm. **h,** IF staining results of NE, MPO, and H3CIT on series sections adjacent to the NETs positive area within the plaque. Scale bar = 100 µm and 20 µm, respectively. **i,** IF staining results of NE and MPO on the series sections adjacent to the area of $H3CIT^+$ $CD68^+$ $Tdtomato^+$ cells. Scale bar = 100 µm and 20 µm, respectively. Each data point represents one mouse. The side of the white star represents the lumen side. White arrows point at the $Tdtomato^+$ H3CIT positive cells within the plaque. For all panels, error bars represent SD. p value was determined by unpaired two-tailed Student's t test. Each experiment was repeated independently 3 times for (c,d,g-i).

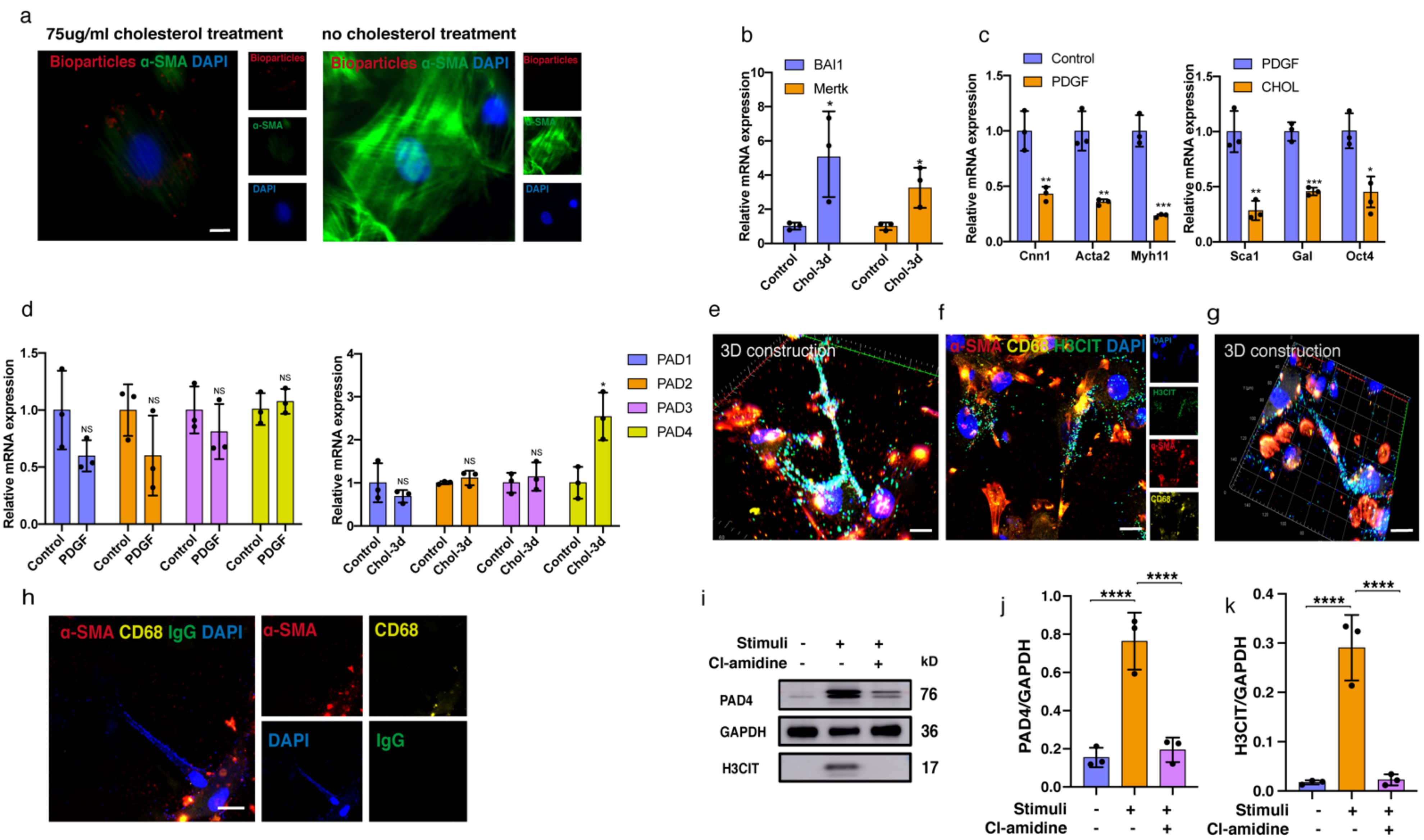

Supplementary Figure 3. Experiments in Vitro Verified RASMCs' Ability of Phagocytosis, mRNA Level's Changes of PAD Family or Stem-related Genes, and ETs Immunostaining of CD68^{+} VSMCs. Related to Figure 2.

a, Cholesterol-loaded RASMCs phagocyted bioparticles and expressed less α-SMA protein stained by IF while no cholesterol-loaded RASMCs phagocyted little. Scale bar = 20 μm. **b,** Cholesterol-loaded RASMCs were highly expressed phagocytic-related genes compared with no cholesterol-loaded RASMCs (n=3 independent experiments). BAI1(*p = 0.042), Mertk (*p = 0.0312). **c,** The relative expression of stem cell-associated genes among RASMCs, PDGF stimulated RASMCs, cholesterol-loaded RASMCs, and PDGF stimulated RASMCs expressed less VSMC marker genes (n=3 independent experiments). Cnn1 (**p = 0.0067), Acta2 (**p = 0.0034), Myh11 (***p = 0.0007), Sca1(**p = 0.0039), Gal (***p = 0.0005), Oct4 (p = 0.0167). **d,** The relative expression of PAD family mRNA between the RASMCs, PDGF stimulated RASMCs, and RASMCs cholesterol-loaded group (n=3 independent experiments). Control vs PDGF (PAD1 p = 0.1336, PAD2 p = 0.1735, PAD3 p = 0.3594, PAD4 p = 0.549) Control vs Chol (PAD1 p = 0.3179, PAD2 p = 0.3297, PAD3 p = 0.5747, PAD4 *p = 0.016). **e-g,** IF staining or 3D-construction results of IF staining of ETs derived from CD68^{+} RASMCs, Scale bar = 20 μm (e). **h,** Normal IgG isotype for negative control. **i,** Cholesterol-induced upregulation of PAD4 expression was reduced after Cl-amidine treatment. Meanwhile, the H3CIT protein's expression was suppressed either. **j&k,** Quantitation of the

immunoblots results of I (n=3 independent experiments). ****P < 0.0001. For all panels, error bars represent SD. p value was determined by unpaired two-tailed Student's t test (b,c,d) or by one-way ANOVA with Bonferroni post-test (j,k). Each experiment was repeated independently 3 times for (a,e-h).

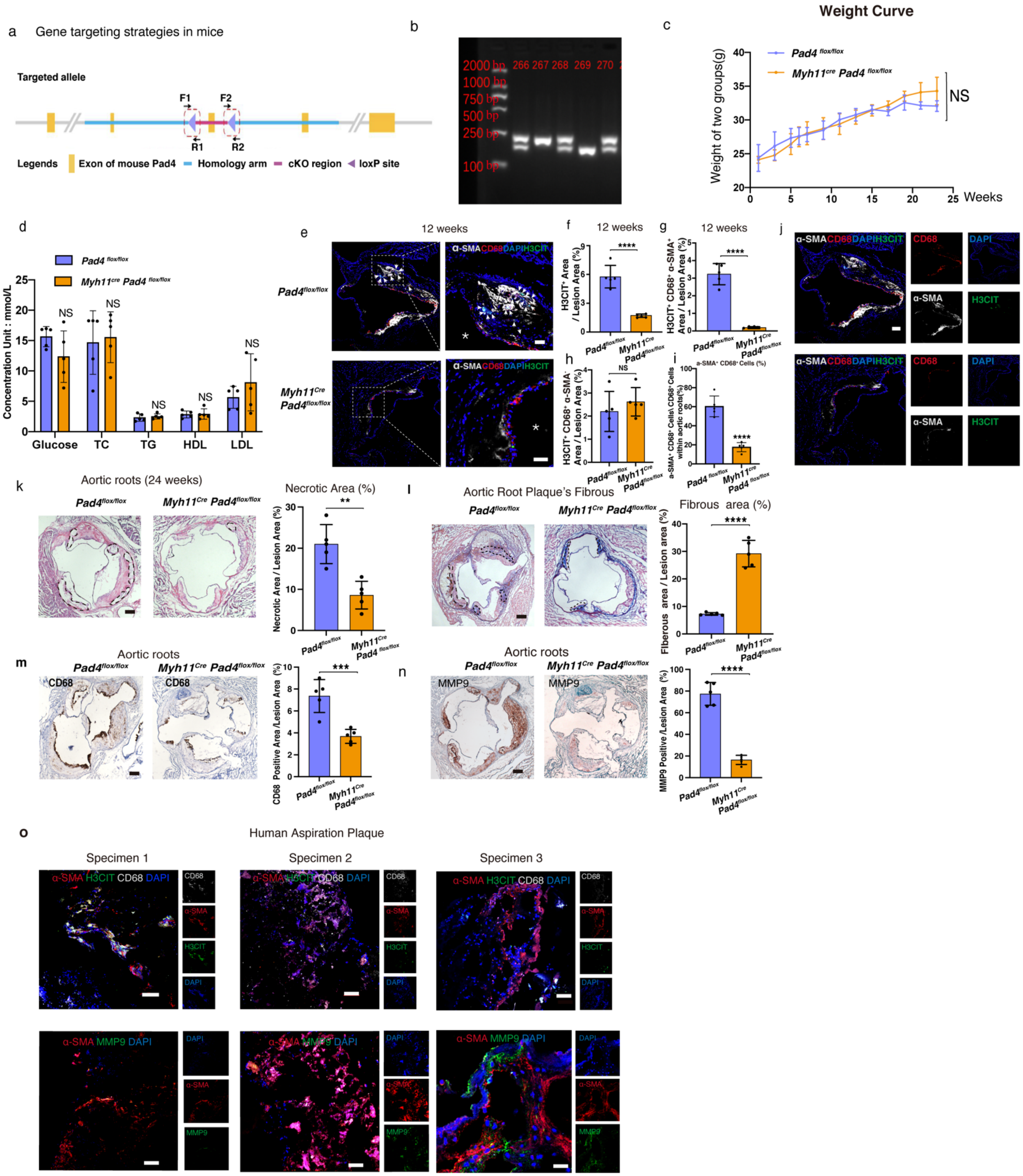
a
Gene targeting strategies in mice
Targeted allele
F1
F2
R1
R2
Legends
Exon of mouse Pad4
Homology arm
cKO region
loxP site
b
2000 bp
1000 bp
750 bp
500 bp
250 bp
100 bp
266 267 268 269 270
c
Weight Curve
Pad4 flox/flox
Myh11cre Pad4 flox/flox
Weight of two groups(g)
Weeks
NS
d
Pad4 flox/flox
Myh11cre Pad4 flox/flox
Concentration Unit : mmol/L
Glucose
TC
TG
HDL
LDL
NS
e
12 weeks
Pad4flox/flox
Myh11Cre Pad4flox/flox
α-SMA CD68 DAPI H3CIT
f
12 weeks
H3CIT+ Area / Lesion Area (%)
g
12 weeks
H3CIT+ CD68+ α-SMA+ Area / Lesion Area (%)
h
H3CIT+ CD68+ α-SMA- Area / Lesion Area (%)
i
a-SMA+ CD68+ Cells (%)
j
α-SMA CD68 DAPI H3CIT
CD68
DAPI
α-SMA
H3CIT
k
Aortic roots (24 weeks)
Pad4flox/flox
Myh11Cre Pad4flox/flox
Necrotic Area (%)
Necrotic Area / Lesion Area (%)
l
Aortic Root Plaque's Fibrous
Fibrous area (%)
Fiberous area / Lesion area (%)
m
Aortic roots
CD68
CD68 Positive Area /Lesion Area (%)
n
Aortic roots
MMP9
MMP9 Positive/Lesion Area (%)
o
Human Aspiration Plaque
Specimen 1
Specimen 2
Specimen 3
α-SMA H3CIT CD68 DAPI
α-SMA MMP9 DAPI

Supplementary Figure 4. Representative Images of Gene Targeting Strategy of *Myh11^{Cre}Pad4$^{flox/flox}$* Mice and Metabolic Indexes of HFD-fed mice Between 2 Groups. Related to Figure 3.

a, Gene targeting strategy of *Myh11^{Cre}Pad4$^{flox/flox}$* mice. **b,** Results of *Myh11^{Cre}Pad4$^{flox/flox}$* mice's genotyping. **c,** A weight change curve between 2 groups. (each group n=5 mice). **d,** Level of glucose, TC, TG, HDL, and LDL in serum between 2 groups of mice (each group n=5 mice). **e,** IF staining in plaque between two groups. Scale bar = 20 µm. **f,** The ratio of H3CIT positive area of total lesion area (each group n=5 mice). ****p < 0.0001 **g,** The ratio of H3CIT$^+$, CD68$^+$, and α-SMA$^+$ area of total lesion area between 2 groups (each group n=5 mice). ****p < 0.0001. **h,** The ratio of H3CIT$^+$, CD68$^{+,}$ and α-SMA$^-$ area in total lesion area between 2 groups (each group n=5 mice). p = 0.4586 **i**, The percentage of CD68$^+$ VSMCs (α-SMA$^+$ CD68$^+$ Cells/CD68$^+$ Cells) in Figure 3I (each group n=5 mice). ****P < 0.0001. **j,** The single fluorescence and merge image of e. Scale bar = 100 µm. **k,** HE staining and ratio of the necrotic core area in the plaque area (each group n=5 mice). Scale bar = 100 µm. The dotted area represents the necrotic area.(**p = 0.0013). **l,** Masson staining and ratio of fibrous area in the plaque area (n=5 mice). Scale bar = 100 µm. The dotted area represents the fibrous area. ****p < 0.0001. **m,** Representative images, and ratio of CD68$^+$ area in plaque area between two groups (each group n=5 mice). Scale bar = 100 µm. ***p value = 0.005. **n,** Representative images, and ratio of the MMP9$^+$ area in plaque area between two groups (each group n=5 mice). Scale bar = 100 µm. Each data point represents one mouse. NS. means no significance. ****p < 0.0001. **o,** IF staining of α-SMA, H3CIT, CD68, and MMP9 in human plaques. Scale bar = 100 µm. For all panels, error bars represent SD. p value was determined by unpaired two-tailed Student's t test. Each experiment was repeated independently 3 times for (b,o).

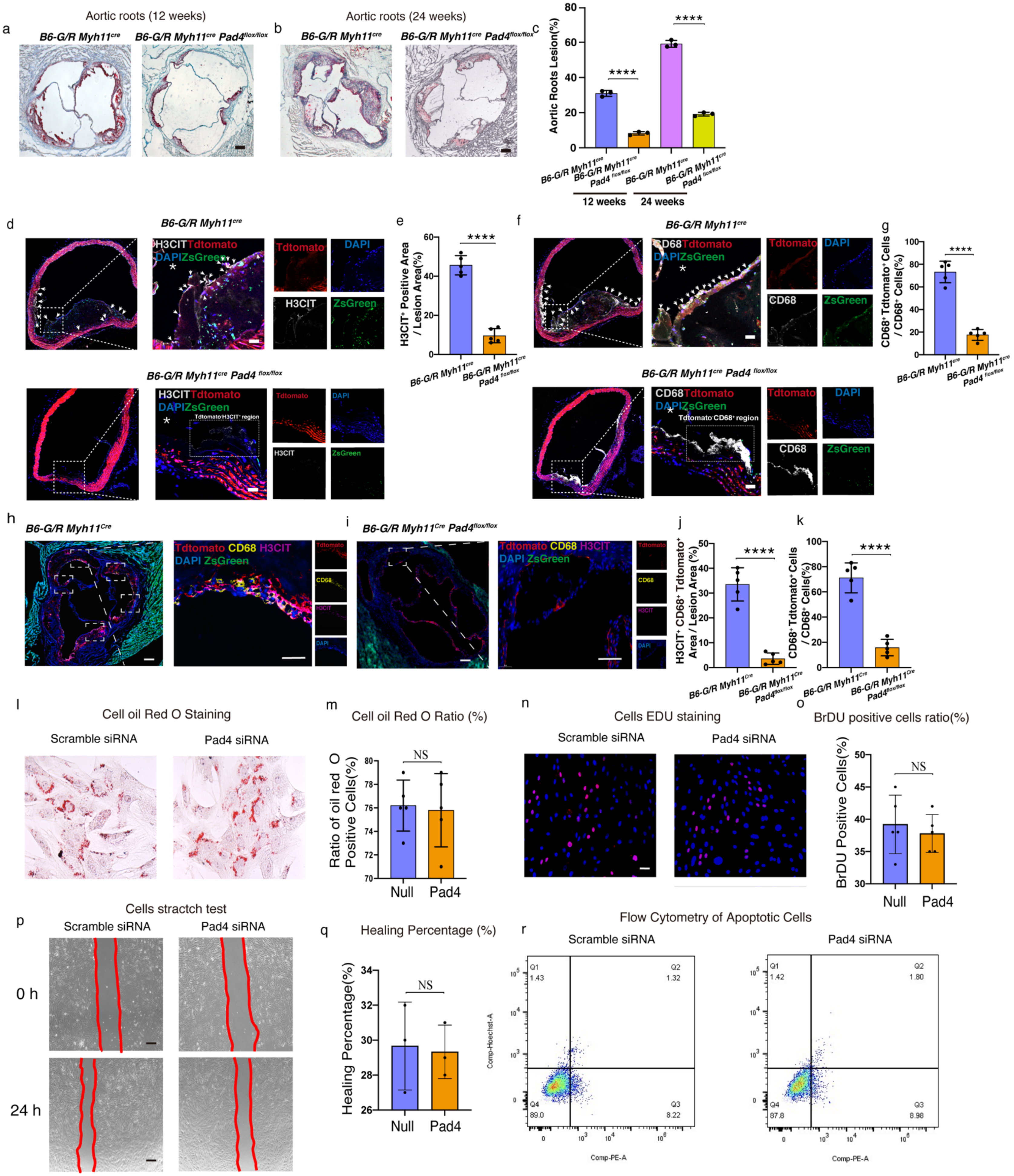

a
Aortic roots (12 weeks)
B6-G/R Myh11cre
B6-G/R Myh11cre Pad4flox/flox
b
Aortic roots (24 weeks)
B6-G/R Myh11cre
B6-G/R Myh11cre Pad4flox/flox
c
Aortic Roots Lesion(%)
****
****
12 weeks
24 weeks
d
B6-G/R Myh11cre
H3CIT Tdtomato DAPI ZsGreen
Tdtomato
DAPI
H3CIT
ZsGreen
B6-G/R Myh11cre Pad4 flox/flox
Tdtomato-H3CIT+ region
e
H3CIT+ Positive Area / Lesion Area(%)
****
f
B6-G/R Myh11cre
CD68 Tdtomato DAPI ZsGreen
CD68
B6-G/R Myh11cre Pad4 flox/flox
Tdtomato-CD68+ region
g
CD68+ Tdtomato+ Cells / CD68+ Cells(%)
****
h
B6-G/R Myh11Cre
Tdtomato CD68 H3CIT
DAPI ZsGreen
i
B6-G/R Myh11Cre Pad4flox/flox
j
H3CIT+ CD68+ Tdtomato+ Area / Lesion Area (%)
****
k
CD68+ Tdtomato+ Cells / CD68+ Cells(%)
****
l
Cell oil Red O Staining
Scramble siRNA
Pad4 siRNA
m
Cell oil Red O Ratio (%)
Ratio of oil red O Positive Cells(%)
NS
Null
Pad4
n
Cells EDU staining
Scramble siRNA
Pad4 siRNA
o
BrDU positive cells ratio(%)
BrDU Positive Cells(%)
NS
Null
Pad4
p
Cells stractch test
Scramble siRNA
Pad4 siRNA
0 h
24 h
q
Healing Percentage (%)
Healing Percentage(%)
NS
Null
Pad4
r
Flow Cytometry of Apoptotic Cells
Scramble siRNA
Pad4 siRNA
Q1 1.43
Q2 1.32
Q4 89.0
Q3 8.22
Q1 1.42
Q2 1.80
Q4 87.8
Q3 8.98
Comp-Hoechst-A
Comp-PE-A

Supplementary Figure 5. Representative Images of Histological Experiments or IF Staining of Atherosclerosis Plaque of *B6-G/R Myh11^{Cre}Pad4$^{flox/flox}$* mice *and B6-G/R Myh11^{Cre}* Mice Respectively. Validation Experiments in Vitro Verified RASMCs' Characteristics After siRNA Intervened. Related to Figure 4.

a-c, Oil red O stained, and relative quantification (each group of each time point, n=3 mice). Scale bar = 100 μm. ****p < 0.0001.**d,** IF staining of H3CIT in plaque between 2 groups of mice. Scale bar = 20μm. **e,** The percentage of H3CIT positive area in the total lesion area (each group n=5 mice). ****p < 0.0001.**f,** IF staining of CD68 in plaque between 2 groups of mice. Scale bar = 20μm. **g,** Ratio of the number of $CD68^+$ $Tdtomato^+$ cells in the number of $CD68^+$ Cells (each group n=5 mice). ****p < 0.0001. **h&i,** IF staining of H3CIT or CD68 in plaque lesions between groups, respectively. Scale bar = 50μm. **j&k,** The percentage of $H3CIT^+$ $Tdtomato^+$ and $CD68^+$ area in the total lesion area, and the percentage of the number of $CD68^+$ $Tdtomato^+$ cells in the total $CD68^+$ Cells (each group n=5 mice). ****p < 0.0001. NS. means no significance. Each data point represents one mouse. **l,** Oil red O staining stimulated RASMCs intervened with siPAD4 or siNC. Scale bar = 20 μm. **m**, Ratio of number of positive RASMCs in the total RASMCs (n=5 independent experiments). p = 0.9824. **n&o,** siPAD4 intervention did not alter RASMCs' proliferation in vitro. Scale bar = 20 μm (n=5 independent experiments). p = 0.5796. **p&q,** RASMCs' ability to migrate in vitro, assessed by cell wound scratch assay (n=3 independent experiments). p = 0.8541. **r,** siPAD4 intervention in RASMCs did not influence their growth ability. NS. means no significance. White arrows pointed at the $H3CIT^+$ or $CD68^+$ $Tdtomato^+$ cells within the plaque. The side of the white star represents the lumen side. The dotted area refers to the $Tdtomato^-$ $CD68^+$ $H3CIT^+$ area. For all panels, error bars represent SD. p value was determined by unpaired two-tailed Student's t test. Each experiment was repeated independently 3 times for p.

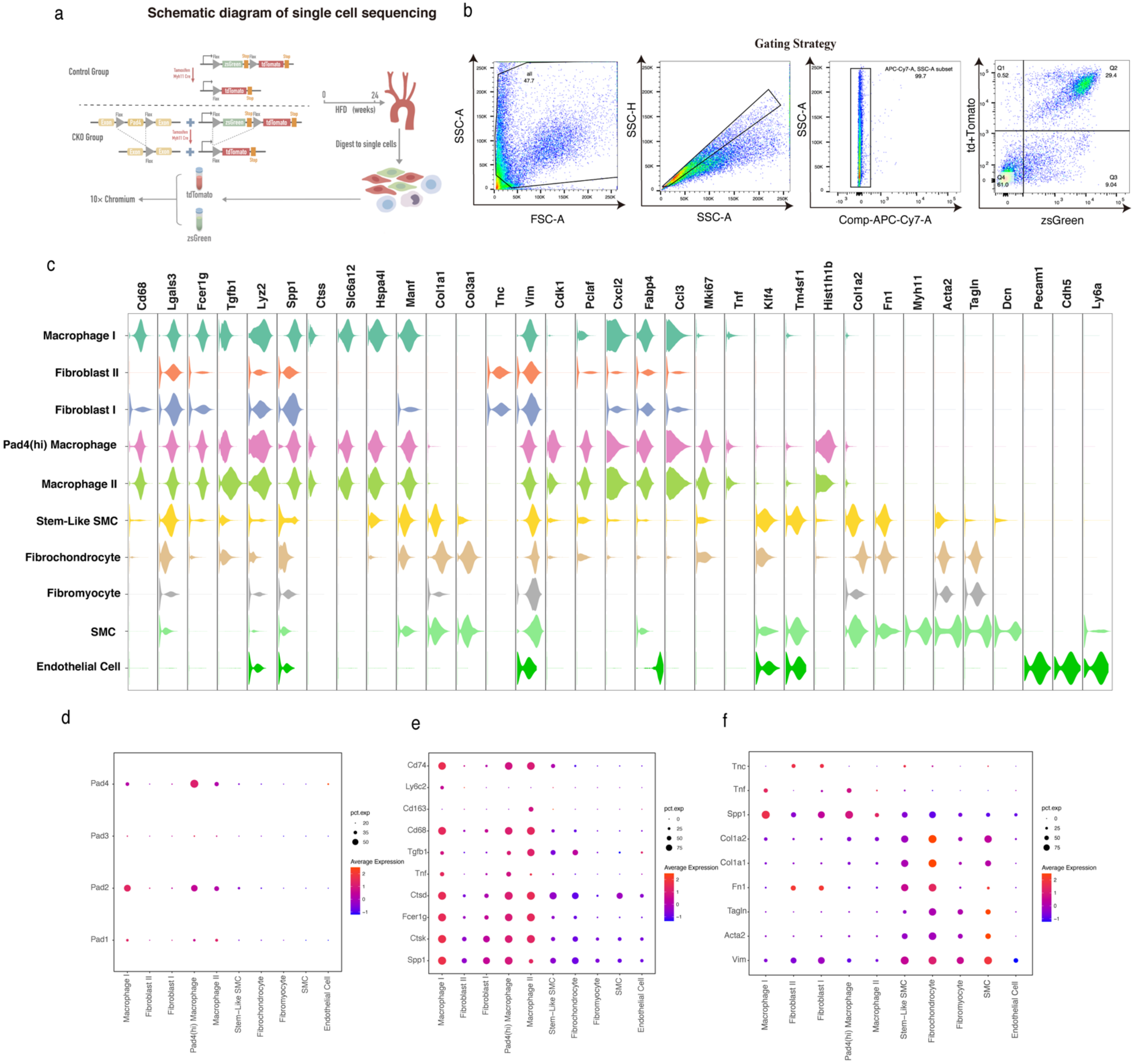
a
Schematic diagram of single cell sequencing
Control Group
CKO Group
HFD (weeks)
Digest to single cells
10× Chromium
tdTomato
zsGreen
b
Gating Strategy
SSC-A
FSC-A
SSC-H
Comp-APC-Cy7-A
td+Tomato
zsGreen
APC-Cy7-A, SSC-A subset
c
Cd68
Lgals3
Fcer1g
Tgfb1
Lyz2
Spp1
Ctss
Slc6a12
Hspa4l
Manf
Col1a1
Col3a1
Tnc
Vim
Cdk1
Pclaf
Cxcl2
Fabp4
Ccl3
Mki67
Tnf
Klf4
Tm4sf1
Hist1h1b
Col1a2
Fn1
Myh11
Acta2
Tagln
Dcn
Pecam1
Cdh5
Ly6a
Macrophage I
Fibroblast II
Fibroblast I
Pad4(hi) Macrophage
Macrophage II
Stem-Like SMC
Fibrochondrocyte
Fibromyocyte
SMC
Endothelial Cell
d
Pad4
Pad3
Pad2
Pad1
pct.exp
Average Expression
e
Cd74
Ly6c2
Cd163
Cd68
Tgfb1
Tnf
Ctsd
Fcer1g
Ctsk
Spp1
f
Tnc
Tnf
Spp1
Col1a2
Col1a1
Fn1
Tagln
Acta2
Vim
Macrophage I
Fibroblast II
Fibroblast I
Pad4(hi) Macrophage
Macrophage II
Stem-Like SMC
Fibrochondrocyte
Fibromyocyte
SMC
Endothelial Cell

Supplementary Figure 6. Representative Images of FACS' Gating Strategy and Cell Grouping Strategy of Single Cell RNA Sequencing. Related to Figure 4.

a, Schematic diagram of scRNA-seq using 10 × Chromium with both Tdtomato^{+} and ZsGreen^{+} cells harvested from aortas of mice fed with HFD for 24 weeks. Arterial tissues (including the aortic arch, ascending aorta, descending artery, brachiocephalic artery, thoracic aorta, and abdominal aorta) with atherosclerotic lesions were isolated and digested to single cells for fluorescence-activated cell sorting (FACS) of Tdtomato^{+} and ZsGreen^{+} cells. Single cells were subsequently loaded to 10 × Chromium for scRNA-seq. **b,** The gating strategy of FACS. **c,** Violin plots showed different markers of total cell clusters. **d-f,** The dot plot showed different marker genes' expression levels and expressed percentages of different clusters in the single cell sequencing results of Figure 4. For all panels, error bars represent SD.

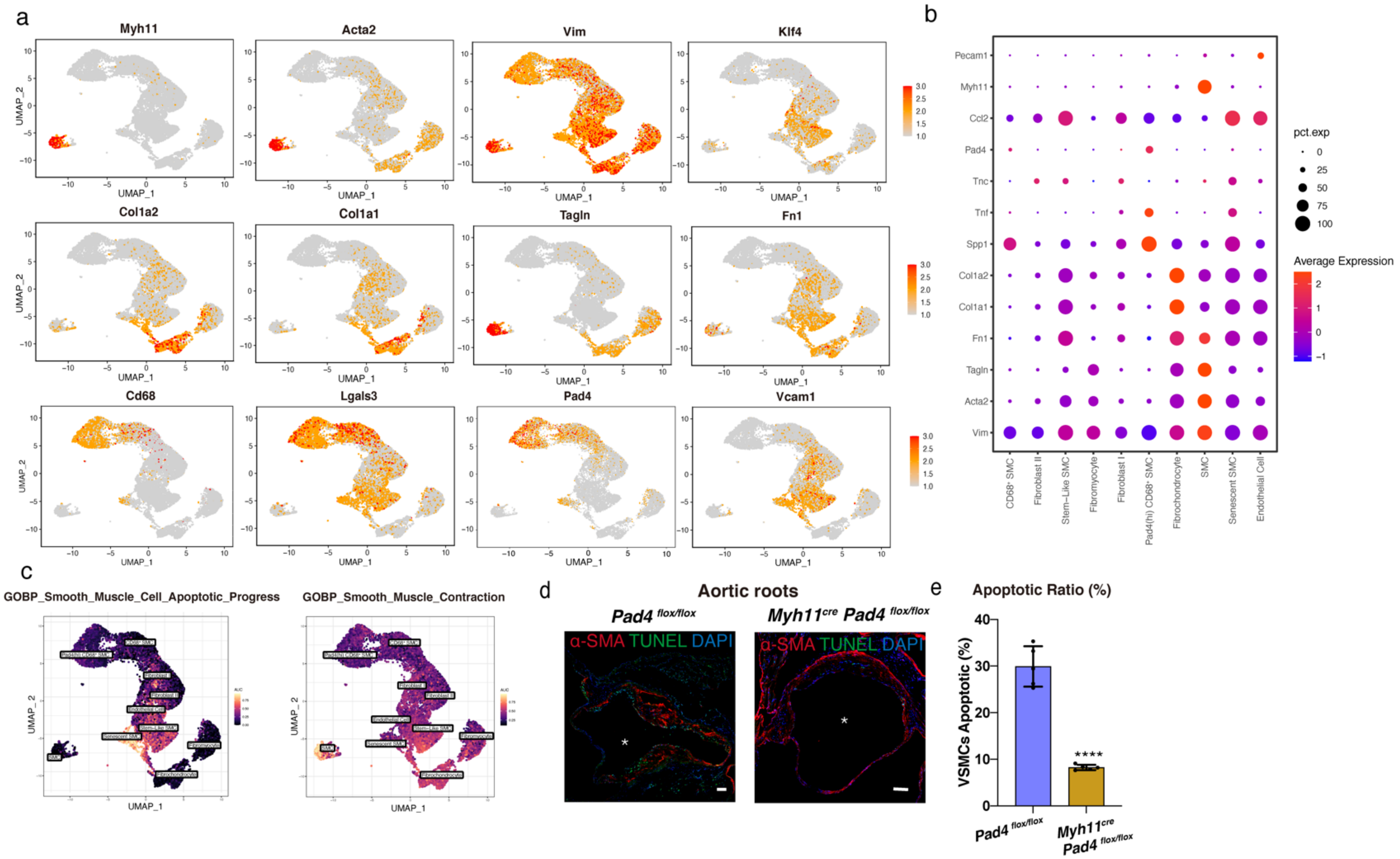

Supplementary Figure 7. Representative Images of Feature Plot of scRNA-seq and TUNEL Experiments. Related to Figure 4.
a, Feature plot showed critical markers of different cell clusters. **b,** Dot plot showed different marker genes' expression and a percentage of different cell clusters in Figure 4k. **c,** The individual cell AUC score overlayed for selected differential canonical pathway activities. **d,** Showed IF results of TUNEL and α-SMA co-staining on the aortic root between two groups (each group n = 4 mice) to assess the ratio of VSMCs apoptosis. **e,** Quantification of apoptotic cells ratio in d (each group n=4 mice). ****$p < 0.0001$. For all panels, error bars represent SD. p value was determined by unpaired two-tailed Student's t test.

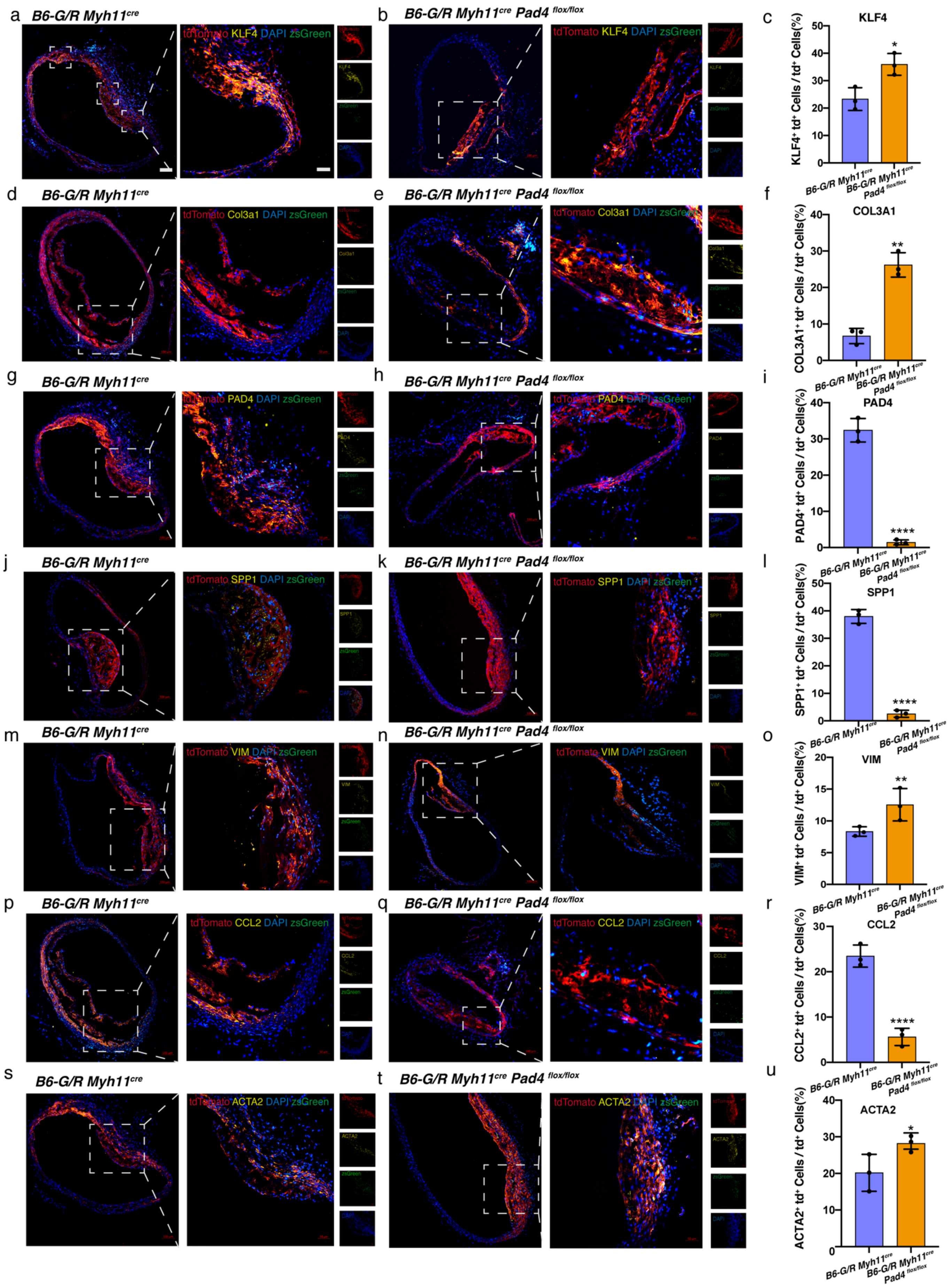

a
B6-G/R Myh11cre
tdTomato KLF4 DAPI zsGreen
b
B6-G/R Myh11cre Pad4 flox/flox
tdTomato KLF4 DAPI zsGreen
c
KLF4
KLF4+ td+ Cells / td+ Cells(%)
d
B6-G/R Myh11cre
tdTomato Col3a1 DAPI zsGreen
e
B6-G/R Myh11cre Pad4 flox/flox
tdTomato Col3a1 DAPI zsGreen
f
COL3A1
COL3A1+ td+ Cells / td+ Cells(%)
g
B6-G/R Myh11cre
tdTomato PAD4 DAPI zsGreen
h
B6-G/R Myh11cre Pad4 flox/flox
tdTomato PAD4 DAPI zsGreen
i
PAD4
PAD4+ td+ Cells / td+ Cells(%)
j
B6-G/R Myh11cre
tdTomato SPP1 DAPI zsGreen
k
B6-G/R Myh11cre Pad4 flox/flox
tdTomato SPP1 DAPI zsGreen
l
SPP1
SPP1+ td+ Cells / td+ Cells(%)
m
B6-G/R Myh11cre
tdTomato VIM DAPI zsGreen
n
B6-G/R Myh11cre Pad4 flox/flox
tdTomato VIM DAPI zsGreen
o
VIM
VIM+ td+ Cells / td+ Cells(%)
p
B6-G/R Myh11cre
tdTomato CCL2 DAPI zsGreen
q
B6-G/R Myh11cre Pad4 flox/flox
tdTomato CCL2 DAPI zsGreen
r
CCL2
CCL2+ td+ Cells / td+ Cells(%)
s
B6-G/R Myh11cre
tdTomato ACTA2 DAPI zsGreen
t
B6-G/R Myh11cre Pad4 flox/flox
tdTomato ACTA2 DAPI zsGreen
u
ACTA2
ACTA2+ td+ Cells / td+ Cells(%)
B6-G/R Myh11cre
B6-G/R Myh11cre Pad4 flox/flox

Supplementary Figure 8. Representative Images of mRNA FISH Test in Aortic Atherosclerosis Plaque and Verification of scRNA-seq Results of Cell Ratio's Changes. Related to Figure 4.

a-c, FISH results of *KLF4* mRNA between groups on the aortic arch plaque's sections of VSMCs tracing mice and related quantification of the ratio of $KLF4^+$ $Tdtomato^+$ cells in total $Tdtomato^+$ cells (each group n=3 mice). *p = 0.019. **d-f,** FISH results of Col3a1 mRNA between groups on the aortic arch plaque's sections of VSMCs tracing mice and related quantification of the ratio of $Col3a1^+$ $Tdtomato^+$ cells in total $Tdtomato^+$ cells (each group n=3 mice). **p = 0.001. **g-i,** FISH results of PAD4 mRNA between groups on the aortic arch plaque's sections of VSMCs tracing mice and related quantification of the ratio of $PAD4^+$ $Tdtomato^+$ cells in total $Tdtomato^+$ cells. (each group n=3 mice). ****p < 0.0001. **j-l,** FISH results of *SPP1* mRNA between groups on the aortic arch plaque's sections of VSMCs tracing mice and related quantification of the ratio of $SPP1^+$ $Tdtomato^+$ cells in total $Tdtomato^+$ cells. (each group n=3 mice). ****p < 0.0001. **m-o,** FISH results of VIM mRNA between groups on the aortic arch plaque's sections of VSMCs tracing mice and related quantification of the ratio of VIM^+ $Tdtomato^+$ cells in total $Tdtomato^+$ cells. (each group n=3 mice). **p = 0.0057. **p-r,** FISH results of CCL2 mRNA between groups on the aortic arch plaque's sections of VSMCs tracing mice and related quantification of the ratio of $CCL2^+$ $Tdtomato^+$ cells in total $Tdtomato^+$ cells. (each group n=3 mice). ****p < 0.0001. **s-u,** FISH results of ACTA2 mRNA between groups on the aortic arch plaque's sections of VSMCs tracing mice and related quantification of the ratio of $ACTA2^+$ $Tdtomato^+$ cells in total $Tdtomato^+$ cells. (each group n=3 mice). *p = 0.0379. The side of the white star represents the lumen side. The dotted area refers to the positive area of the FISH test. Scale bar = 100 μm and 50 μm, respectively. For all panels, error bars represent SD. p value was determined by unpaired two-tailed Student's t test.

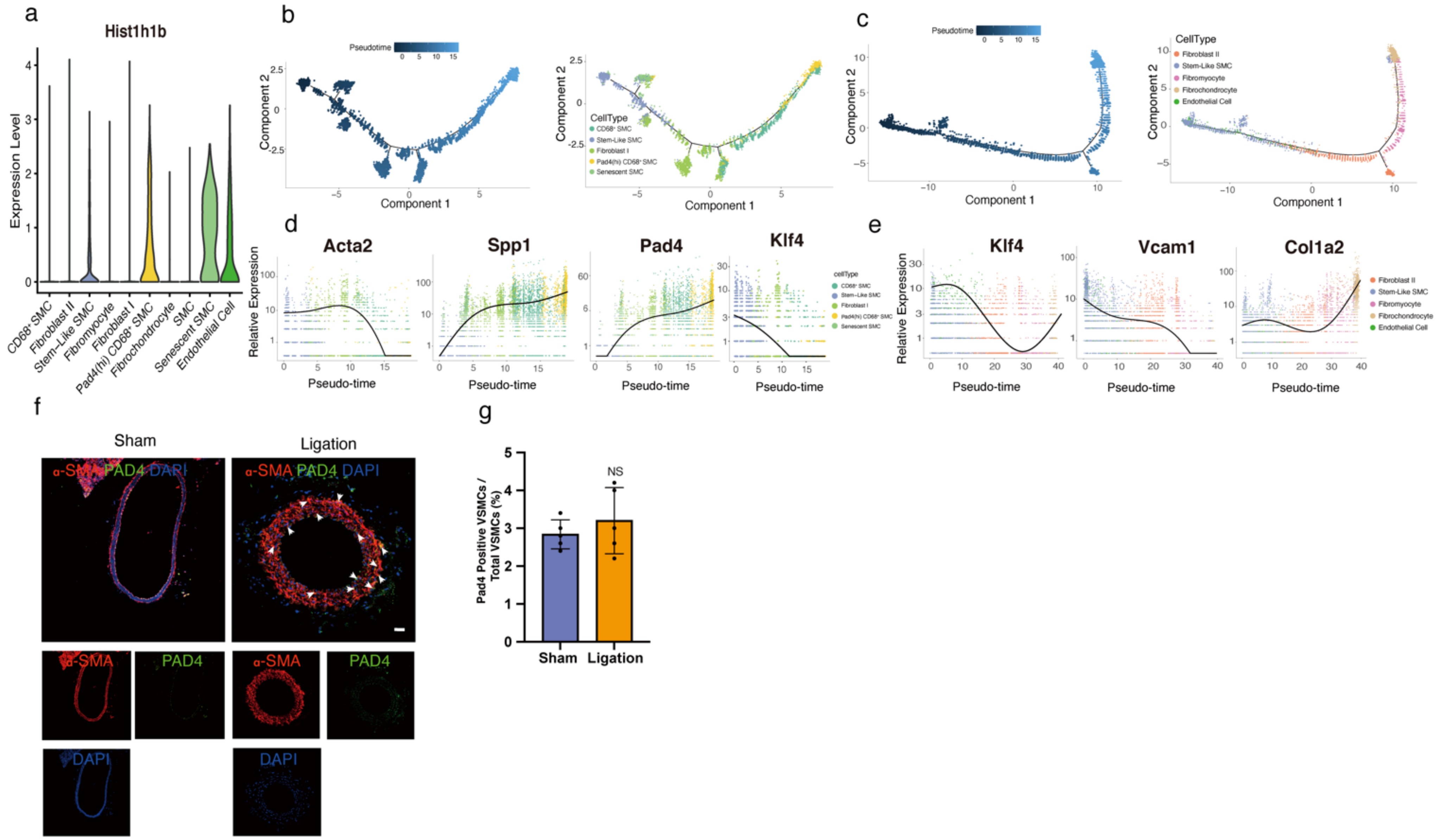

Supplementary Figure 9. Representative Images of Cell Trajectory Analysis and IF Staining of PAD4 within Atherosclerosis Plaque from 2 Groups of Mice. Related to Figure 4.

a, Violin plots showed Hist1h1 b′s expression level of different clusters. **b-c,** Monocle analysis of the harmful-like or beneficial populations indicated pseudo time directionality and cell type, the colors of cell type were consistent with the cell in Figure 4k. **d,** Genes' expression tendencies of Acta2, Spp1, Pad4, and Klf4 among harmful cell populations. **e**, Genes' expression tendencies of Klf4, Vcam1, and Col1a2 among beneficial cell populations. **f,** The IF staining of PAD4 and α-SMA in the carotid artery of sham mice or carotid artery ligated mice. Scale bar = 100 μm. **g,** The Quantification of PAD4 positive VSMCs of total VSMCs of f. (each group n=5 mice). p = 0.5053. NS. Means no significance. For all panels, error bars represent SD. p value was determined by unpaired two-tailed Student’s t test.

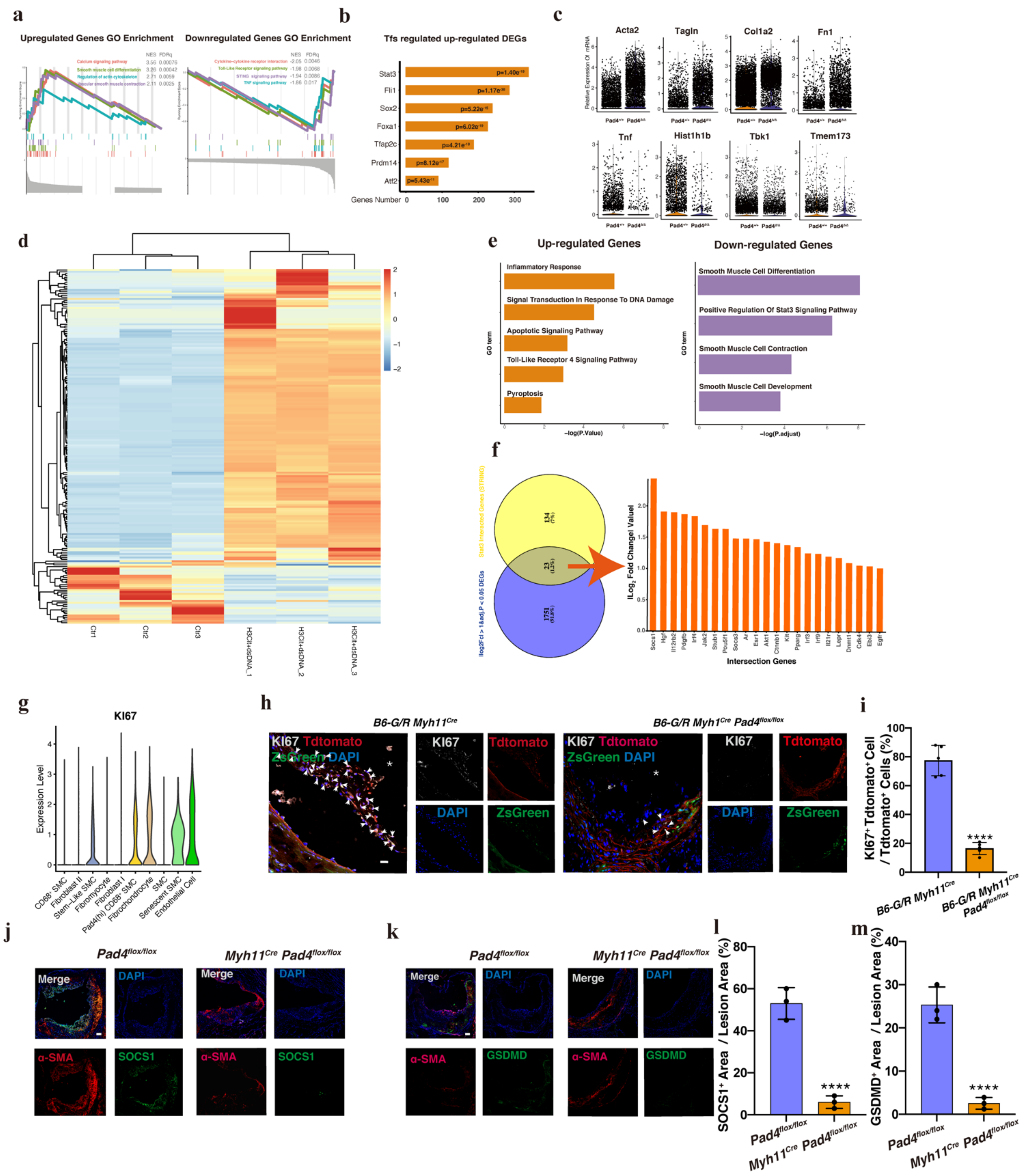
a
Upregulated Genes GO Enrichment
Downregulated Genes GO Enrichment
b
Tfs regulated up-regulated DEGs
Stat3
Fli1
Sox2
Foxa1
Tfap2c
Prdm14
Atf2
Genes Number
c
Acta2
Tagln
Col1a2
Fn1
Tnf
Hist1h1b
Tbk1
Tmem173
d
Ctrl1
Ctrl2
Ctrl3
H3Cit+dsDNA_1
H3Cit+dsDNA_2
H3Cit+dsDNA_3
e
Up-regulated Genes
Down-regulated Genes
Inflammatory Response
Signal Transduction In Response To DNA Damage
Apoptotic Signaling Pathway
Toll-Like Receptor 4 Signaling Pathway
Pyroptosis
Smooth Muscle Cell Differentiation
Positive Regulation Of Stat3 Signaling Pathway
Smooth Muscle Cell Contraction
Smooth Muscle Cell Development
GO term
-log(P.Value)
-log(P.adjust)
f
Intersection Genes
g
KI67
Expression Level
h
B6-G/R Myh11Cre
B6-G/R Myh11Cre Pad4flox/flox
KI67 Tdtomato
ZsGreen DAPI
i
KI67+ Tdtomato+ Cell / Tdtomato+ Cells (%)
****
j
Pad4flox/flox
Myh11Cre Pad4flox/flox
Merge
DAPI
α-SMA
SOCS1
k
GSDMD
l
SOCS1+ Area / Lesion Area (%)
m
GSDMD+ Area / Lesion Area (%)

Supplementary Figure 10. Representative Images of DEGs analysis based on sc-RNAseq, Bulk RNA-seq, and IF Staining Results of $Ki67^+$ $Tdtomato^+$ Cells in Atherosclerosis Plaque of 2 Groups of Mice. Related to Figure 5.

a, GSEA analysis results of upregulated genes and down-regulated genes and related NES or FDRq value of enriched pathway. **b,** Predicted TFs regulating the upregulated DEGs, regulating DEGs number and adjust p.value of TFs were marked. **c,** Violin plot of major DEGs comparing two groups of sc-RNAseq results. **d,** Heatmap showed different gene expression patterns among groups of bulk RNA-sequencing results. **e,** GO-enrichment results of upregulated or down-regulated DEGs screened from bulk RNA-seq data. **f,** The Venn diagram showed integrated DEGs with STRING predicted genes regulated STAT3 signaling pathway, and the screened genes ranked as the absolute value of $\log_2$ fold change value of bulk RNA sequencing results. **g,** Violin plot showed the expression level of Ki67 of different cell clusters in the results of single-cell RNA sequencing. **h,** IF staining showed the $Ki67^+$ $Tdtomato^+$ VSMCs in the aortic plaque of VSMCs lineage tracing mice between two groups. Scale bar = 100 μm. **i,** The Quantification of the percentage of the number of $Ki67^+$ $Tdtomato^+$ VSMCs in the total number of $Tdtomato^+$ VSMCs between 2 groups (each group n = 5 mice). The side of the white star represents the lumen side. White arrows point at the $Ki67^+$ $Tdtomato^+$ Cells within the plaque. ****p < 0.0001. **j&k,** The single fluorescence and merge image of SOCS1 and GSDMD within aortic roots, respectively. Scale bar = 100 μm. **l&m,** The Quantification of the ratio of SOCS1 and GSDMD positive area in plaque lesion area between 2 groups (each group n=3 mice). ****p < 0.0001. For all panels, error bars represent SD. p value was determined by unpaired two-tailed Student's t test.

Supplementary Table 1

Gene (Rat)	Sequence
**GAPDH**	Forward: 5′-GACATGCCGCCTGGAGAAAC-3′<br>Reverse: 5′-AGCCCAGGATGCCCTTTAGT-3′
**MAC2**	Forward:5'-ATCCTGCTACTGGCCCCTTT-3′<br>Reverse:5'- -GCGATGTCGTTCCCTTTCTT-3'
**ABCA1**	Forward 5'-CCTAAGCATTATCAAGGAGGGAAG-3'<br>Reverse 5' -AGAGATGACAAGGAGGACGGAAG-3'
**CD68**	Forward 5'-TCCTTCACGGAGACACCT-3'<br>Reverse 5' -GGCTGGGAACCATTAGTC-3'
**CX3CR1**	Forward 5'-AGCTGCTCAGGACCTCACCAT-3′<br>Reverse 5'- GTCATATGCAGGAACTCTGGG-3'
**ACTA2**	Forward 5'-ATCTTTTCCATGTCGTCCCAGTTG-3'<br>Reverse 5'-GAGAAGCCCAGCCAGTCG-3'
**MYH11**	Forward 5'-AATGCCCTCAAGAGCAAACTCAG-3'<br>Reverse 5'-TCCCGAGCATCCATTTCTTCTTCA-3'
**CNN1**	Forward 5'- ACACTTTAACCGAGGTCCTGCCTA-3'<br>Reverse 5'- CTTGAGGCCATCCATGAAGTTGCT-3'
**PAD1**	Forward: 5′-CACAGGCAAGGTGAAGAAAGG-3′<br>Reverse: 5′-TGTTTGTAGTTGGAGAGGGAGG-3′
**PAD2**	Forward: 5′-TCCTGAAAGAGGTGAAGAACCTG-3′<br>Reverse: 5′-TACTGGGAAGCCTTTGTGAGG-3′
**PAD3**	Forward: 5′-TTGGCTTGTGCTTCCTATGGT-3′<br>Reverse: 5′-GTAGTGAGGGTGTGAAATAGTCTG-3′
**PAD4**	Forward: 5′-GCTGGGAAGGATCAGAGCAC-3′<br>Reverse: 5′-GGGAGTCTTCGTGCTTAGGG-3′
**GAL**	Forward: 5'-TGGAAGTGGAGGAAGGGAGACTAGG-3'<br>Reverse: 5' GGGATGCCAGGCAGGCTGTC 3'
**SCA-1**	Forward: 5'-AACCATATTTGCCTTCCCGTCT-3'<br>Reverse: 5'-CCAGGTGCTGCCTCCAGTG-3'
**OCT4**	Forward: 5'-CGAGGCCTTTCCCTCTGTTCCT-3'<br>Reverse: 5'-TCTCTTTGTCTACCTCCCTTCCTTGC-3'

Supplementary Table 2. The baseline characteristics of the patients

	ACS (n=4)
**Age**	69.5 ± 6.5
**Male/Female**	2 / 2
**Hypertension**	4 (100%)
**Stroke**	2 (25%)
**Smoking history**	1 (25%)
**Diabetes**	2 (50%)

Supplementary Table 3. The sequence of siPAD4 and siNC

	Sense	Anti-sense
si-PAD4	CUCCCAAAGACUUCUUUGATT	UCAAAGAAGUCUUUGGGAGTT
si-NC	UUCUCCGAACGUGUCACGUTT	ACGUGACACGUUCGGAGAATT
