# Supplementary material for: Extracellular traps from activated vascular smooth muscle cells drive the progression of atherosclerosis
Source: Nat Commun. 2022 Dec 6;13:7500. doi: 10.1038/s41467-022-35330-1 (PMC9723654; doi:10.1038/s41467-022-35330-1)
Supplement: Supplementary file 3 — Reporting Summary [file 41467_2022_35330_MOESM3_ESM.pdf]

nature portfolio

Corresponding author(s): Wenhui Peng

Last updated by author(s): Nov 11, 2022

Reporting Summary

Nature Portfolio wishes to improve the reproducibility of the work that we publish. This form provides structure for consistency and transparency in reporting. For further information on Nature Portfolio policies, see our Editorial Policies and the Editorial Policy Checklist.

Statistics

For all statistical analyses, confirm that the following items are present in the figure legend, table legend, main text, or Methods section.

n/a	Confirmed	
☐	☒	The exact sample size (*n*) for each experimental group/condition, given as a discrete number and unit of measurement
☐	☒	A statement on whether measurements were taken from distinct samples or whether the same sample was measured repeatedly
☐	☒	The statistical test(s) used AND whether they are one- or two-sided<br>*Only common tests should be described solely by name; describe more complex techniques in the Methods section.*
☐	☒	A description of all covariates tested
☐	☒	A description of any assumptions or corrections, such as tests of normality and adjustment for multiple comparisons
☐	☒	A full description of the statistical parameters including central tendency (e.g. means) or other basic estimates (e.g. regression coefficient) AND variation (e.g. standard deviation) or associated estimates of uncertainty (e.g. confidence intervals)
☐	☒	For null hypothesis testing, the test statistic (e.g. *F*, *t*, *r*) with confidence intervals, effect sizes, degrees of freedom and *P* value noted<br>*Give P values as exact values whenever suitable.*
☒	☐	For Bayesian analysis, information on the choice of priors and Markov chain Monte Carlo settings
☒	☐	For hierarchical and complex designs, identification of the appropriate level for tests and full reporting of outcomes
☒	☐	Estimates of effect sizes (e.g. Cohen's *d*, Pearson's *r*), indicating how they were calculated

Our web collection on statistics for biologists contains articles on many of the points above.

Software and code

Policy information about availability of computer code

Data collection	ZEN 2009 light edition (Carl Zeiss), BD FACSDiva, Light Cycler 96 version 1.1,Agilent 2100 Bioanalyzer,STAR aligner (2.4.0i),“HTseq” (https://htseq.readthedocs.io, version 0.6.0),Aperio Versa 8 tissue imaging system (Leica).Procreate (V4.5.3).
Data analysis	GraphPad Prism (version 8.0), FlowJo (version 10.07, USA), Image J (V2.10), Seurat v3.1.1, DoubletFinder(Version 2.02), Monocle (Version 3.0), Dbi R package (org.Mm.eg.db), R packages clusterProfiler (4.0) , enrichPlot (1.0) and ggplot2 (Version 4.0), R package AUCell(2.0), DESeq2 （Version 2.0), KnockTF (http://www.licpathway.net/KnockTF/index.html). The code using for analyzing the data was deposited in the Github according to the link (https://github.com/ZhaiMing0/Extracellular-traps-from-activated-VSMCs).

For manuscripts utilizing custom algorithms or software that are central to the research but not yet described in published literature, software must be made available to editors and reviewers. We strongly encourage code deposition in a community repository (e.g. GitHub). See the Nature Portfolio guidelines for submitting code & software for further information.

Data

Policy information about availability of data

All manuscripts must include a data availability statement. This statement should provide the following information, where applicable:

- Accession codes, unique identifiers, or web links for publicly available datasets
- A description of any restrictions on data availability
- For clinical datasets or third party data, please ensure that the statement adheres to our policy

All the data supporting the findings of this study are available within the article and its Supplementary Information files and from the corresponding author upon reasonable request. FASTQ files and expression matrices from mouse scRNA-seq data or rat RNA-seq data are available from the NCBI Gene Expression Omnibus (GEO) database under the accession number GSE197073 and GSE197074 respectively (Publicly released). The source data underlying all Figures and Supplementary Figures are provided as a Source Data file. The rat reference genome NCBI Rnor6.0 (https://www.ncbi.nlm.nih.gov/assembly/GCF_000001895.5/). The GSEA using the reference gene sets selected GOc5.all.v7.1.symbols.gmt.(http://www.gsea-msigdb.org/gsea/msigdb/human/collections.jsp#C5).

Human research participants

Policy information about studies involving human research participants and Sex and Gender in Research.

Reporting on sex and gender	The human research participant is not a sex/gender related research and we collected the aspiration plaque from 2 male and 2 female patients after the written informed consent was collected from patients or their relatives.
Population characteristics	Pathological records were reviewed, and formalin-fixed paraffin-embedded coronary plaque were obtained from the archives of the Department of Pathology, The Tenth People's Hospital, Tongji University. Four cases with ACS including ST-segment elevation myocardial infarction were enrolled. The aspiration plaque was obtained from 2 males and 2 females who accepted aspiration throm-bectomy, average age is 69 years. All the patients had hypertension, 2 patients had a previous stroke, 1 patients had a smoking history, 2 patients had diabetes.
Recruitment	The four human coronary plaque was obtained from an aspiration Export catheter (Medtronic). The written informed consent was collected from patients or their relatives. The plaque was fixed with 4% paraformaldehyde for 10 min. Paraformaldehyde-fixed plaque samples were embedded in paraffin blocks and cut into 5 µm thick sections for further analysis. These results indicated that the 4 patients could not completely represent the real population, and there may be selection bias. But we randomly selected these patients, there was no subjective intervention. Moreover, these 4 patients average aged about 69y and combined with risk factors (smoking, male, hypertension, stroke, diabetes), so they were high-risk patients for ACS. Therefore, we believe that they are representative to a certain extent. Of course, we also hope that a larger study will verify our results in the future
Ethics oversight	The study was approved by the Ethical Committee of Shanghai Tenth People’s Hospital (permit number: 22KN22).

Note that full information on the approval of the study protocol must also be provided in the manuscript.

Field-specific reporting

Please select the one below that is the best fit for your research. If you are not sure, read the appropriate sections before making your selection.

☒ Life sciences ☐ Behavioural & social sciences ☐ Ecological, evolutionary & environmental sciences

For a reference copy of the document with all sections, see nature.com/documents/nr-reporting-summary-flat.pdf

Life sciences study design

All studies must disclose on these points even when the disclosure is negative.

Sample size	No statistical methods were used to pre-determine sample sizes. Based on the literature and our previous studies, for each experiment we used at least n = 3 replicates to calculate the statistical values for each analysis.
Data exclusions	No data were excluded from this study.
Replication	As for the scRNAseq, we performed one experiment of scRNA-seq of pooled Tdtomato+ cells and ZsGreen+ cells sorted from atherosclerotic aortas of B6-G/R Myh11CrePad4foxl/flox mice and B6-G/R Myh11Cre mice (A total of 12 littermate mice, each group is 6 mice were used in this experiment) because the cost prohibitive, but we verified the scRNA-seq results by using mFISH, and is at least 3 independent experiments. Other experiments were repeated at least three times to reliably support the conclusions stated in the manuscript.
Randomization	For all experiments, age/sex-matched mice were used to control for potential co-variates. Mice from the same cage were used when possible. When mice were purchased for experiments, they were maintained in the same room at least 2 weeks to allow acclimatisation. For in vivo treatment experiments, mice from the same cage were randomly selected for different treatment. For genetic deletion

experiments, littermate mice were selected based on their genotype. For in vitro experiments, cells from the same biological sample (rat) were plated into separate wells as multiple replicates and the wells were randomly selected for different treatment.

Blinding

For histology quantification (lipid/CD68/H3CIT/α-SMA/LY6G/MMP9/GSDMD/H&E/MASSON), images were anonymously renamed to enable blind analysis. For other analyses, blinding was not required since experimental readouts were quantitative.

Reporting for specific materials, systems and methods

We require information from authors about some types of materials, experimental systems and methods used in many studies. Here, indicate whether each material, system or method listed is relevant to your study. If you are not sure if a list item applies to your research, read the appropriate section before selecting a response.

Materials & experimental systems

n/a	Involved in the study
☐	☒ Antibodies
☒	☐ Eukaryotic cell lines
☒	☐ Palaeontology and archaeology
☐	☒ Animals and other organisms
☒	☐ Clinical data
☒	☐ Dual use research of concern

Methods

n/a	Involved in the study
☒	☐ ChIP-seq
☐	☒ Flow cytometry
☒	☐ MRI-based neuroimaging

Antibodies

Antibodies used

The following antibodies were used for immunofluorescence and immunohistochemistry staining:
anti-Citrullinated Histone 3 (ab5103, Abcam, 1:100),
anti-α-smooth muscle actin (α-SMA, ab7817, Abcam, 1:200),
anti-CD68 (NB100-683, Novus, 1:100),
anti-LY6G (127601, Biolegend, 1:100),
anti-MPO (AF3667, R&D, 1:100),
anti-PAD4 (ab2148, Abcam, 1:100),
anti-GSDMD (20770, Proteintech, 1:100),
and anti-Ki-67 (ab15580, Abcam, 1:100). N
ormal isotype IgG (sc2027, Santa Cruz, 1:100) ;
Alexa Fluor 647-conjugated goat anti-rabbit (A-21244, Invitrogen, 1:200),
Alexa Fluor 647-conjugated goat anti-rat (A-21247, Invitrogen, 1:200),
Alexa Fluor 488-conjugated goat anti-rabbit (A-11008, Invitrogen, 1:200),
Alexa Fluor 488-conjugated donkey anti-rat (A-21208, Invitrogen, 1:200),
Alexa Fluor 594-conjugated goat anti-rat (A-11007, Invitrogen, 1:200),
Alexa Fluor 594-conjugated donkey anti-rabbit (R37119, Invitrogen, 1:200),
and Alexa Fluor 594-conjugated goat anti-mouse (A-11005, Invitrogen, 1:200)
The following antibodies were used for WB:
anti-Citrullinated Histone 3 (ab5103, Abcam, 1:100),
anti-α-smooth muscle actin (α-SMA, ab7817, Abcam, 1:200),
anti-CD68 (NB100-683, Novus, 1:100),
anti-LY6G (127601, Biolegend, 1:100),
anti-MMP9 (ab38898, Abcam, 1:100) ,
anti-GAPDH (60004-1-Ig, Proteintech, 1:5000)
anti-Beta Actin (66009-1-Ig, Proteintech,1:5000)
anti-MYD88 (SC-74532, Santa Cruz, 1:600),
anti-TLR4 (SC-10741, Santa Cruz, 1:500),
anti-pSTAT3 (9145, Cell Signaling Technology, 1:1000),
anti-Stat3 (9139, Cell Signaling Technology, 1:1000),
anti-SOCS1 (ab9870, Abcam,1:800),
and anti-STING (ab288157, Abcam, 1:1000).
For antibodies used in the flow cytometry:
live/dead fixable viability stain 780 (565388, BD Horizon),
extracellular DNA dye (SYTOX Blue, S11348, Invitrogen),
CD16/32 antibody (101320, Biolegend, 1:100),
anti-LY6G (127641, Biolegend, 1:100),
anti-CD68 (137016, Biolegend, 1:100),
anti-α-SMA (ab208844, Abcam, 1:100).

Validation	Commercially available antibodies were validated by the manufacturers; anti-mouse antibodies were validated by using mouse tissues (bone marrow cells, peritoneal macrophages or splenocytes or mouse cell lines (J774A.1).Anti rat antibodies were validated by using the rat tissues (aorta, skin) or rat cell lines (A7r5). Purified antibodies for histology were optimised using mouse tissue sections prior to use for experiments.

Animals and other research organisms

Policy information about studies involving animals; ARRIVE guidelines recommended for reporting animal research, and Sex and Gender in Research

Laboratory animals	A total number of 25 male 8 weeks aged Ldlr-/- mice, a total number of 30 male 8 weeks aged Pad4flox/flox Myh11Cre mice, a total number of 30 male 8 weeks aged Pad4flox/flox mic, and a total number of 30 male 8 weeks aged B6G/R Myh11Cre mice, and a total number of 30 male 8 weeks aged B6-G/R Myh11CrePad4flox/flox mice participated in our research. A total number of 25 male Sprague-Dawley rats (200–250 g, 8 weeks) were used for extracting RASMCs for vitro experiments.
Wild animals	The study did not involve wild animals.
Reporting on sex	As for our study, the male Ldlr -/- ; the Myh11CrePad4flox/flox mice , the male Pad4flox/flox mice, the male B6-G/R Myh11CrePad4flox/flox, and the male B6-G/R Myh11Cre mice were used in our study. Since the gene encoding Myh11-cre enzyme is located on the Y chromosome, in order to ensure the consistency of the studies, we used male mice to models of atherosclerosis and conduct experimental studies. The methods used for our assigning sex is that according to the mice genitals' characteristic at 6 weeks after them born. For Sprague-Dawley rats, we used the male rats to extract RASMCs for conducting vitro experiments to ensure the consistency of our studies.
Field-collected samples	The study did not involve samples collected from the field.
Ethics oversight	Animal procedures were approved by the Animal Care and Use Committees of Shanghai Tenth People's Hospital.

Note that full information on the approval of the study protocol must also be provided in the manuscript.

Flow Cytometry

Plots

Confirm that:

- [x] The axis labels state the marker and fluorochrome used (e.g. CD4-FITC).
- [x] The axis scales are clearly visible. Include numbers along axes only for bottom left plot of group (a 'group' is an analysis of identical markers).
- [x] All plots are contour plots with outliers or pseudocolor plots.
- [x] A numerical value for number of cells or percentage (with statistics) is provided.

Methodology

Sample preparation	Mice were sacrificed by cervical dislocation, and vasculature was flushed with PBS to completely remove blood. Arterial tissues, enriched with atherosclerosis plaque, including the aortic arch, ascending aorta, descending artery, brachiocephalic artery, thoracic aorta, and abdominal aorta, were isolated, minced to about 1 mm piece, and placed into tissue dissociation solution (130-110-201, Miltenyi Biotec). After incubation at 37 °C for 30 min in a magnetic stirrer, cell suspensions were filtered through 70m strainers and centrifuged at 400× g for 5 min. The cell pellets were washed once with 0.2% FBS in PBS for FACS or Flow Cytometry.
Instrument	BD LSR-II
Software	FlowJo (V10.0.7, USA)
Cell population abundance	Viable cells in all analyses were over 85% based on fsc/ssc. Td+ /α-SMA+ cells were over 80% purity in aortic detected by FACS/ Flow Cytometry.
Gating strategy	The Fluorescence Minus One Control were used to define the boundaries between positive and negative cell populations in mutiple fluorochromes panels. Viable cells were selected based on fsc/ssc. The following gating strategies were used: CD68+ VSMC cells were gated as CD68+ α-SMA+. Neutrophils cells were gated as Ly6g+ . ETs positive cells were used extracellular DNA dye.

- [x] Tick this box to confirm that a figure exemplifying the gating strategy is provided in the Supplementary Information.
